# Stomatitis of Pregnant, and Nursing Women

**Published:** 1853-04

**Authors:** David Hutchinson


THE NORTH-WESTERN
MEDICAL AND SURGICAL JOURNAL.

NEW SERIES.

VOL. I. APRIL, 1853. No. 12.

### ORIGINAL COMMUNICATIONS.

ART. I.—*Stomatitis of Pregnant, and Nursing Women.* By DAVID HUTCHINSON, M. D.

THE literature of this disease is very scanty, and scattered over the American medical periodicals of the last twelve years; although it has but recently attracted attention, I am of opinion that its existence is of considerable date. Abercrombie in his inimitable work on the stomach and bowels, details a case, which, from the description, must have been this disease, and which he ultimately cured, with a decoction of logwood. It is his 115 case, page 356, second ed.

Stomatitis Materna, is certainly a misnomer, as the sore mouth, is only a sequence of the previous state of system, although the sore mouth is generally the first symptom that attracts the attention of the patient, for it sometimes comes on very suddenly, yet in all the cases that I have observed, derangement of the digestive organs, to a greater or less extent, existed anterior to the ulcerations of the mouth, and, the buccal ulcerations generally bore a proportionate relation to the gastric derangement; the greater the gastric disturbance, the more extensive and difficult to heal, were the ulcers in the mouth. Dr. Brainard has described the disease as ulcerations of the vagina, connected with the states of utero gestation, these ulcerations of the vagina, invariably alternate, with the ulcerations of the mucous membranes of the mouth and small intestines, and exist in every case where the disease continues for any length of time, these destructive ulcerations being the result of the abnormal condition of the nutritive system; all my cases were troubled with flatulence, pyrosis, and irregular bowels, costiveness alternating with diarrhœa, which continued prominent symptoms throughout the affection. Dr. Brainard says that the ulcerations are the effect of a general cause, connected with an anemic condition of system.

Dr. Shields in an essay on the disease in the Western Journal of Medicine and Surgery, attributes it entirely to lactation. I beg leave to differ with him on this point, as the most inveterate case that I have ever seen, occurred two weeks before delivery. Dr. Wood says "that the cause is some influence, exerted on the system, by the advanced state of pregnancy and lactation; what this influence is, is unknown, as it does not seem to be connected with any pre-existing condition of health, or character of constitution. I have never seen a case, but there had previously existed an anemic condition of system, and it is most apt to occur in those who have previously suffered from hemorrhages, leucorrhea, and other debilitating affections; I have known it to follow enlargement of the spleen, produced by ague. Might not the cause be found in the chemical changes that take place in the blood during pregnancy? It is a fact established by the researches of chemists, that the blood, especially in the latter stages of utero-gestation, undergoes great alterations. According to Simon, the density, both of the defibrinated blood and of the serum is diminished—the water, the fibrin, and the phosphorized fat are increased, while the corpuscles and the albumen are diminished. It is also well known, that the specific gravity of the blood is diminished during pregnancy. I found the saliva, that flows so abundantly in this affection, highly albuminous; now, we ask, is it not probable that this condition of the blood in a female, previously anemic, would induce deranged sensibility of the gastric branch of the par vagum nerve, and perhaps also of the sympathetic, producing an altered condition of the gastric secretions, and consequent indigestion, giving rise to flatulence, some eructations, burning at epigastrium, and various gastric disturbances of the stomach? or might not this state of blood be brought about by the abnormal nutrition of the system that the disturbed condition of the stomach must produce? because, when the first processes of digestion are interfered with, the subsequent processes are imperfect; consequently, the formation of food into the requisite chyle globules, and subsequently blood globules are also interfered with, hence there is a consumption of the blood globules, and a consequent increase of fibrin. Hence, there obtains a similar condition of blood in this disease, as in atrophy scrofulosis, tuburculosis, and other cachectic states. The destructive ulcerations that are set up in the mucous membranes of the mouth, small intestines, and vagina are the result of this altered state of blood, and perverted nutrition. The state of the kidneys and urinary secretion, also demands attention. In all the cases that came under my observation, there existed great paucity of urine for some time before the sore mouth made its appearance, with a frequent disposition to urinate, which passed in small quantities at a time, with pain and smarting, and the urine, after standing a few hours, threw down a very copious precipitate. It is well known that a pathological relation exists between the kidneys and stomach, and the gastric secretions are much influenced by their inefficient action, and when the urinary salts are unable to escape by their natural outlet, they will seek egress by the salivary and gastric glands, and by the liver, and occasion a very considerable amount of derangement in these organs. I found saline cathartics and alkalies to remedy this condition of the kidneys. It being produced by the ill-conditioned fibrin of the blood. It is well known that salines lessen the acidity of the urine, and decrease the fibrin of the blood, and I observed that the condition of the mouth and stomach invariably improved with the re-establishment of the function of the kidneys, and I doubt not but that iodide potass. which appear to be adapted to the mild form of this affection, has been very useful by increasing the depurating function of these organs. We have in this affection: 1st, Abnormal or depraved nutrition, depending on derangement of the digestive organs, existing before the ulcerations take place in the mouth, bowels, or vagina.

2nd, An altered state of the blood, viz.: an increase of water, fibrin and phosphorized fat, and a decrease of albumen and corpuscles, produced by pregnancy, lactation, and depraved nutrition.

3rd, As a sequence, destructive ulcerations of the mucous surfaces of the mouth, intestines and vagina, and a wasting of the tissues.

Should this pathology be correct, the treatment is obvious. I will detail a few cases, which will illustrate this affection.

There are two forms, or grades of the disease, acute and subacute. The first form generally makes its appearance, either before or shortly after delivery. The second is more apt to occur, some weeks or months after. In the subacute, the pulse is but little affected, the patient does not loose her appetite for food, she remains able to go about, although feeble, and suffers from weakness and nervous symptoms: such cases, I have generally relieved by remedies addressed to the correction of the condition of the stomach, and five grs. iodide potass. three times per day.

Case 1.—Mrs. W. had ulcers on the sides of the tongue, mouth very tender, child three months old, and was laboring under dysentery in a mild form, she was relieved in a few days, by correcting the dysenteric symptoms, and Iodide potass. five grs. three times a day.

My second case was similar to the above, and was speedily relieved by a similar treatment.

My third case occurred in a lady that had the disease in the subacute form, in a previous nursing. This time she had suffered much during the latter months of utero-gestation from gastric derangement, acid eructations, and costiveness, alternating with diarrhœa, scanty high colored urine, with a frequent disposition to urinate, which was attended with smarting and burning sensations. While in this condition she was attacked two weeks before delivery, with a scalding sensation in the mouth, extending down the œsophagus to the stomach, a profuse discharge of hot burning saliva, loss of appetite and of taste, tongue very red around the edges, and in patches on the dorsum. On the second day, the pain in the mouth and jaws became distracting and intolerable, discharge of hot saliva from mouth very profuse, pulse full and tense, bowels constipated, urinary secretion very scanty; 24 oz. of blood was abstracted from the arm, which gave her great comfort, by relieving the intolerable pain in the mouth and jaws, and she took a saline drought, which brought away very dark fœtid evacuation, and reduced the febrile and inflammatory condition of system. The inside of the cheeks, gums, edges, and underpart of the tongue, became covered with small ulcers, with a red, fiery surface around them. Nit. argent. and also muriatic acid, as a lotion, were applied to the ulcers, without any apparent beneficial effects. She also took iodide potass. for several days, without benefit. The stomach continued deranged, acid eructations, and a hot burning sensation at epigastrium. The iodide potass. was laid aside, and she was given an oz. aqua calcis, three times a day, and saline aperients. The tart. potass. and soda, to keep the bowels soluble, which corrected the state of the stomach, the urinary secretion became more abundant, and by the application of the nit. argent. as a lotion, the ulcers began to heal and the mouth speedily improved. A few days after delivery, the sore mouth returned. The tongue assumed the same aspect as before, ulcers formed on the sides and underpart of the tongue, and inside of the cheeks, a febrile condition of system, of the hectic character existed, much gastric derangement, acid eructations and burning at the epigastrium, coated tongue, impaired appetite, and scanty high-colored urine, with painful micturition. The urine, on standing a few hours, threw down a copious precipitate. She was again given saline, aperients, tart. potass. and soda, aqua calcis, and the lotion of nit. argent. to ulcers, when, after five days' treatment, the state of the stomach and bowels became corrected, and the state of the mouth again began to improve rapidly, and here I noticed, that the lotion nit. argent. had no influence on the ulcers in the mouth, until the condition of the stomach was corrected.

In two weeks after she recovered from the second attack, the disease again returned, mouth exceedingly tender, edges and underpart of the tongue covered with ulcers, tongue furred, scanty, pink, red-colored urine, throws down a heavy precipitate on standing, frequent pulse, hectic, exhausting nocturnal perspirations, loss of appetite, pyrosis, flatulence, discharges from bowels hot and excoriating, flow of savila abundant; emaciated fast, and began to sink low; this time aqua calcis failed to relieve the burning at epigastrium. Bicarb. soda was given, which gave much relief.

Having seen Dr. Evans's remarks on the use of cod liver oil in this affection, I thought it a case well adapted to it, and as my patient was sinking fast, being unable to sit up any at all for several weeks, and with difficulty turn herself in bed, I thought best to give her tonics, at the time that she was taking the oil. Accordingly, she was placed on the following: cod liver oil—a table spoon full thrice a day, in a little brandy—sub nit. bismuth, grs.v. thrice a day—quinine, grs.ij. thrice a day—solut. bicarb. soda, ad libitum, as a drink. The bismuth and soda, acted almost as a charm, in correcting the state of the stomach, so that from this treatment in four days she was much improved. The tongue and mouth lost their fiery color, the ulcers began to heal, the nocturnal sweats ceased, the febrile condition of system became less, the appetite and strength began to return. The treatment was continued three weeks, when she was quite restored, and continued to nurse her child, a fine, healthy, stout boy. It required aperients, to keep the bowels soluble. The salines appeared to produce the best influence, and especially the tartrate of soda and potass.; the urinary secretion being always more abundant after their action, and a consequent improvement of the stomach and mouth. All that I have read on the subject recommend weaning; I think it unnecessary, as the sore mouth will frequently continue after weaning, unless the condition of the nutritive organs is corrected by appropriate treatment. A lady applied to me a few months ago, she had undergone various treatments, had weaned her child, and yet the mouth did not get well; she suffered from gastric derangement, ulcers in the mouth, and vagina, bad appetite and a feeble state of health; she had taken cod liver oil, and refused to take any more, on account of its disagreeableness. I gave her sub. nit. bismuth, gentian and soda, which improved her digestive organs, and she speedily recovered. How the bismuth produces its effects in this affection is a little uncertain, but it is probably by changing the sensibilities of the par vagum nerve of the sympathetic.

The following is a pleasant mode for its administration: sub nit. bismuth, ʒj.—tinct. ginger, tinct. cordam. gum accacia, aa. ʒj. —aqua, ℥viij.—shake well, from a tea to a table spoonful after eating or before.

I have observed in those cases in which the disease returns, after the mouth is once relieved, that, during the intervals of the attacks, ulcerations in the vagina take place, and when they disappear in the vagina, they will again appear in the mouth, and vice versa.

I found animal food, best adapted to the condition of my patients' stomachs, when the tenderness of the mouth would permit them to eat it.

I think much might be done by way of prophylaxis, by attending strictly to the condition of the stomach in the latter stages of pregnancy. In conclusion, I would beg leave to request some member of the profession, that is accustomed to chemical manifestations, to analyze the blood and urine in this affection, and let the results of the analysis be known to the *profession.*

